# Developing policy and regulation for the safer use of artificial intelligence technologies for mental health in Australia

**DOI:** 10.1177/00048674261434403

**Published:** 2026-03-30

**Authors:** Kylie Maidment, Helen Christensen, Jack Lattimore, Jill M. Newby, Alexis E. Whitton

**Affiliations:** 1 Black Dog Institute, University of New South Wales, Sydney, NSW, Sydney 2052, NSW; 2 School of Psychology, University of New South Wales, Sydney, NSW, Sydney 2052, NSW

**Keywords:** Artificial Intelligence, Mental health, Policy, Regulation, Recommendations

## Abstract

Artificial intelligence (AI) is emerging as a powerful tool for improving mental health research and care, offering opportunities for early intervention, personalised treatment, ongoing monitoring, and enhanced capacity for the mental health workforce. In Australia, however, the integration of AI into mental health systems is hindered by substantial knowledge, policy, and regulatory gaps. This paper outlines two urgent priorities for the safe and effective use of AI in mental health: (1) closing knowledge gaps, and (2) developing robust policy and regulatory frameworks. We outline emerging opportunities, including AI-driven technologies that could improve affordability, accessibility, and treatment outcomes, as well as tools to strengthen the mental health workforce, alongside key risks such as inadequate regulation, insufficient monitoring or reporting of adverse events, perpetuation of bias in research, data privacy and security concerns, and lack of human oversight. We provide 10 recommendations to guide the safer adoption of AI for mental health in Australia. These include the creation of a National AI in Mental Health Expert Advisory Group, evidence-based national guidelines, expanded data collection on AI use in mental health, Australian-led research that considers priority populations, development of Australian databases for training AI models, targeted investment in AI technologies that can support an under-resourced mental health workforce, creation of AI mental health literacy resources for the Australian public, and regulations that hold developers and providers accountable for the safety of their technologies.

To date, no policy change, innovative treatment, or funding increase has been able to counter the rising rate of mental health conditions in Australia (Australian Institute of Health and Welfare [AIHW], 2021). Rapid advances in artificial intelligence (AI), however, offer new opportunities to support mental health prevention, early intervention, and treatment (Mutebi and Gardiner, 2025). Emerging AI-enabled tools may provide cost-effective support for both mental health professionals and the public, including applications for clinician training (Holmes et al., 2025) and personalised treatments selection (Squires et al., 2023).

As these technologies are already widely accessed by consumers outside formal healthcare pathways, the critical policy question is no longer whether AI will be used in mental health care, but how it can be governed to be safer than existing unregulated consumer AI tools and ad-hoc uses. Assessing the safety, effectiveness, and appropriateness of AI within the Australian mental health system is therefore urgent. Safety in this context is multidimensional, encompassing clinical appropriateness, emotional harm, privacy, equity, transparency, and is perceived differently by consumers, clinicians, services, regulators and developers.

This scrutiny is particularly timely for three reasons. First, the Australian Government has committed $1 billion over the next four years to expand mental health services and build the mental health workforce capacity (Butler, 2025). Arguably, this investment is unlikely to meet its goals without leveraging the capabilities of AI to improve access to treatment (Habicht et al., 2024), reduce administrative burden (Wilson et al., 2023) and expand training of mental health professionals and other workers (Chaszczewicz et al., 2024). Second, AI is already being used for mental health support in Australia. Surveys indicate that Australians are using generative AI tools (e.g., ChatGPT) for “quick support” and as a “personal therapist” (Cross et al., 2025), with analyses of recent online content identifying “therapy/companionship” as the most frequent use cases (Zao-Sanders, 2025). Third, many of these technologies were not designed for mental health care and fall outside current Australian regulatory frameworks, as they do not meet the Therapeutic Goods Administration (TGA) definition of a medical device.

Young Australians are rapid adopters of new technologies. Following the release of ChatGPT in November 2022, an exploratory study of 576 Australians aged 14-26 found that 65% had used a generative AI chatbot by 2023 (Denejkina, 2023). By 2024, this had risen to 84% among 15–24-year-olds, with ChatGPT the most popular tool. As a result, AI technologies are increasingly likely to shape youth mental health care (Marshall et al., 2025). The ability to discern between high-quality information and disinformation will be important skills for future generations.

Generative AI systems, including large language models (LLMs), produce new content based on patterns learned from large text corpa and are commonly accessed via chatbots. While such systems can be used to obtain mental health information and support, regulatory oversight is closely tied to intended purpose. At the time of writing (January 2026), no AI-based chatbots are supported by clinical trials and registered on the Australian Register of Therapeutic Goods (ARTG) to “diagnose, treat or cure mental health conditions”. Chatbots developed by mental health clinicians and grounded in psychological science (e.g., Therabot (Heinz et al., 2025) and Woebot (Campellone et al., 2025a)) are not readily accessible in Australia.

Regulatory complexity arises when general-purpose AI tools are used beyond their intended purpose. Widely available chatbots such as Claude and ChatGPT were not designed to diagnose or treat mental health conditions and do not meet the TGA definition of a medical device. However, emerging evidence indicates that people are using these tools for mental health support, creating potential risks.

A 2023 Medical Journal of Australia perspectives article characterised Australia’s AI health policy environment as “embryonic”, noting that existing frameworks focus primarily on AI embedded in clinical devices rather than general-purpose systems (Coiera, Verspoor and Hansen, 2023). Given the rapid expansion of AI technologies and their increasing use for mental health support, addressing these knowledge and regulatory gaps is now a matter of urgency.

Accordingly, this paper outlines two key considerations for the safer use of AI technologies in mental health in Australia: persistent knowledge gaps and risks associated with gaps in policy and regulation. We assume that AI may contribute to improving mental health care, but that these two considerations must be addressed for the benefits to be realised.

## Opportunities and knowledge gaps in the safe use of AI for mental health

### Knowledge gaps

AI-enabled technologies may expand access to mental health care across five phases: screening and triage, treatment, monitoring, clinical education and population-level prevention (Ni and Jia, 2025). While these applications remain at an early stage, significant knowledge gaps must be addressed to understand their capabilities. Current and emerging opportunities requiring further investigation are summarised in Table 1.

### Risks associated with policy and regulatory gaps

Australia currently lacks clear policies and guiding principles for the use of AI in mental health care. These gaps undermine efforts to develop effective mental health technologies and to support the Australian mental health workforce. They also create a cascade of risks spanning public health, research quality, clinical care, data governance, and real-world deployment.

#### Public health

Publicly available generative AI tools are not regulated, yet they can produce outputs that are indistinguishable from human therapists (Hatch et al., 2025). These tools lack genuine empathy (Blease and Rodman, 2024), and raise ethical and safety concerns (Saeidnia et al., 2024). Many were not designed for mental health purposes and fall outside current regulatory frameworks.

#### Research

Weak regulatory guidance has also contributed to a lack of high-quality research in this area. Studies are often limited by non-standardised methods of evaluation, non-validated clinical scales, small sample sizes, and lack of replication (Hua et al., 2025). Few studies focus on individuals with diagnosed mental health conditions, instead targeting general wellbeing, which typically places such technologies outside existing regulatory requirements (Kolding et al., 2025).

#### Clinical treatment

In clinical settings, AI introduces ethical challenges related to confidentiality, informed consent, and algorithmic bias. Advanced AI chatbots may exhibit sycophantic behaviour, agreeing with users even when they are incorrect (Wei et al., 2024). Biases in AI systems may exacerbate existing mental health disparities related to gender, cultural background or socioeconomic status (Omar et al., 2025).

#### Data privacy, security, and sovereignty

Mental health data are among the most sensitive personal information, yet current privacy laws may not adequately protect individuals from risk (Office of the Australian Information Commissioner [OAIC], 2024a). Recent data breaches (OAIC, 2024b) highlight the vulnerability of large datasets and the potential harms of misuse. Data sovereignty is particularly critical for First Nations people, yet many AI systems scrape large volumes of data without the consent of data owners. Such practices undermine Indigenous data sovereignty and risk producing outputs that are unrepresentative of First Nations populations and potentially harmful (Perera et al., 2025).

#### Deployment without oversight

Finally, recent incidents illustrate the risks of deploying AI-based (and even simpler non-AI automated systems) without proper human oversight. For example, the Robodebt program (2016-2019) used an automated algorithm to recover debt without accounting for individual circumstances like financial hardship, disproportionately harming marginalised communities already at increased risk of poor mental health (Jobberns and Guihot, 2024). More recently, the NSW Department of Education discovered that Microsoft Teams had automatically enabled collection of students’ biometric data (e.g., face and voice) for a month without consent (Taylor, 2025), highlighting the risks associated with integrating AI tools into public systems without adequate governance.

## Current solutions to address risk

Given the current gaps in knowledge, policy, and regulation, it is important to consider what measures, if any, are currently in place to mitigate risks associated with the use of AI in mental health contexts. This section briefly outlines existing Australian legislation and regulatory mechanisms that may apply, and highlights limitations that must be addressed.

### Mental health professionals using AI

There is currently no AI-specific legislation governing the use of AI in clinical care in Australia. Mental health professionals must instead comply with existing laws, such as the *Privacy Act* (1988), and must be guided by regulations and professional standards outlined by the TGA, Australian Health Practitioner Regulation Agency (AHPRA), and the Australian Commission on Safety and Quality in Health Care (ACSQHC). The Australian Government has acknowledged that existing frameworks may not adequately address harms associated with high-risk AI applications, including those in healthcare settings. Existing frameworks also provide limited guidance on important issues such as algorithmic decision-making, model updates, or accountability for AI-generated outputs. The National AI Safety Institute, announced in late 2025, is intended to provide independent expert oversight and advice to support the safe development and use of AI in Australia, and may help to address some of these gaps.

### AI in medical devices

In Australia, regulatory requirements apply when AI is used in medical devices, defined as tools used for the “diagnosis, prevention, monitoring, prediction, prognosis, treatment, or alleviation of disease, injury or disability.” The TGA has recently recommended a review to assess whether new technologies such as AI digital scribes are medical devices (TGA, 2025). The TGA regulates AI used in medical devices under the Software as a Medical Device (SaMD) framework. However, there are circumstances where such AI tools may be exempt, particularly where they follow established clinical practice guidelines. For example, an AI chatbot that administers and scores the PHQ-9, recommends consulting a GP based on established thresholds, and explicitly references the RANZCP guidelines, would likely fall outside formal regulation. This illustrates how current frameworks can leave substantial areas of AI-enabled mental health activity unregulated.

### Unregulated technologies

At the time of writing, there are no Australian policies or regulations governing the use of general-purpose AI chatbots (e.g., ChatGPT) by the public for mental health support. Although these technologies were not designed for mental health purposes, they are increasingly used in this way (Cross et al., 2025), particularly by vulnerable populations (Habicht et al., 2024; Ayre, Cvejic and McCaffery, 2025). Many of these tools are developed outside Australia, with data stored on overseas servers, raising privacy and security concerns. As a result, Australians may be exposed to significant risks when using unregulated AI for mental health support, yet there are currently no systematic mechanisms to monitor or mitigate these harms.

## Recommendations

AI presents unique and rapidly evolving risks, and the current period represents a critical window in which early policy and regulatory decisions will shape its long-term impact on mental health care. Regulatory inaction at formative stages of technological adoption has historically had enduring consequences. For example, Section 230 of the 1996 *Communication Decency Act* in the United States stated that “no provider or user of an interactive computer service shall be treated as the publisher or speaker of any information provided by another information content provider.” This effectively shielded social media platforms from legal responsibility over harmful content, contributing to downstream public health and mental health harms that proved difficult to address retrospectively.

We argue that a similar pattern of regulatory lag in relation to unregulated and general-purpose AI risks exposing the Australian public to comparable harms. In response, we propose ten recommendations that address current knowledge gaps and regulatory and policy shortcomings, with the aim of supporting safer use of AI in mental health contexts.

### A Strengthen national governance and decision-making capacity

#### 1 Establish a National AI in Mental Health Expert Advisory Group to support timely, evidence-informed governance and decision-making

This independent, multidisciplinary group should include representatives from mental health organisations, lived experience and relevant professional bodies. Its role would be to support policy and system-level decisions where formal regulation is limited or still evolving. This group would:

a) provide rapid, independent advice to Government on the benefits, risks, and appropriate use of emerging AI-based mental health technologies;
b) develop national guidance outlining acceptable and unacceptable uses of AI in mental health contexts;
c) conduct rapid reviews to inform time-sensitive policy and regulatory decisions;
d) define a minimum set of indicators or decision thresholds to assess alignment with Australian mental health standards (e.g., privacy, cultural safety, clinical oversight);
e) advise health services and professional bodies on safety, quality, and adverse-event monitoring related to AI-use.

#### 2 Establish ongoing national monitoring of public use of AI for mental health support

Commit to regular, nationally representative surveys to track how Australians use AI for mental health information, companionship, therapy and support, including perceived benefits and harms. These data should directly inform policy responses and public education efforts.

### B Close critical evidence gaps to support policy and regulation

#### 3 Target research investment to address high-priority regulatory and safety questions

Direct Australian research investment toward clearly defined regulatory, safety and clinical decision-making needs related to the use of AI in mental health care. Priority should be given to studies that resolve areas of regulatory uncertainty and inform practical decisions about when, where, and for whom AI tools can be used safely. This should include clinical trials to assess efficacy and effectiveness of AI tools, evaluation of ethical and safety risks, adverse events and reporting, algorithmic bias, and mechanisms for informed consent.

#### 4 Develop representative Australian datasets to support equitable AI for mental health

The outputs of LLMs and other generative AI systems are highly dependent on the representativeness of their training data. To support the equitable use of generative AI in mental health contexts, Australia requires well-governed datasets that reflect local clinical practice, cultural contexts and population diversity. Investment should support development of secure Australian mental health data assets that enable training and fine-tuning of AI systems for mental health applications. These data initiatives should incorporate strong privacy protections, clear governance and access controls, and be designed in partnership with people with lived experience, including Aboriginal and Torres Strait Islander peoples and other priority populations. The Medical Research Future Fund is ideally placed to support the development of these national data assets given its mandate to fund large-scale, translational infrastructure that underpins safe and effective health innovation.

#### 5 Prioritise research with and for priority populations

Support research on the impacts of AI on the mental health of young people and priority populations, including Aboriginal and Torres Strait Islander people, culturally and linguistically diverse communities, and LGBTQIA+ people. While AI may be a transformative tool for First Nations communities, there are significant concerns regarding the digital divide (Bentley et al., 2024), data sovereignty and cultural sensitivity (Perera et al., 2025). Accordingly, research should include co-design with lived experience, attention to digital exclusion, and strong protections for data sovereignty and cultural safety.

### C Invest in AI technologies to support an under-resourced mental health workforce

#### 6 Invest in evidence-based AI tools that reduce administrative burden

Prioritise the adoption of validated AI technologies that support mental health professionals with documentation, scheduling and other administrative tasks, where these tools demonstrably improve efficiency without compromising safety or professional judgment. Procurement should be contingent on evidence of benefit, and safeguards for data security and clinician accountability.

#### 7 Develop and evaluate AI-enabled training tools for the mental health workforce

Support the development and evaluation of generative AI-based training tools that simulate clinical interactions to support skill development, supervision, and continuing professional education. These tools may be used to provide realistic practice scenarios (e.g., Socratic questioning, assessment, responding to challenging presentations) within a controlled environment (Holderried et al., 2024; Holmes et al., 2025).

#### 8 Establish clear training and development pathways for AI use in mental health practice

Develop national guidance to support workforce capability and accountability in the use of AI. This should include:

a) ongoing education on the risks, limitations, and potential benefits of AI in mental health settings; and
b) evidence-based guidance to support the selection and appropriate use of AI tools in clinical practice, including clarity regarding professional responsibility and liability. Professional development pathways may incorporate competency assessment, accreditation processes, peer support, and supervision.

### D Improve public AI mental health literacy

#### 9 Develop national AI mental health literacy resources for the Australian public

Invest in coordinated public education initiatives to improve understanding of how AI is used for mental health support. This includes resources that outline the potential benefits, limitations and risks of using AI for mental health purposes, information that promotes realistic expectations of AI tools and their capabilities, and clear guidance that directs users toward evidence-based options.

### E Establish accountability for developers and providers

#### 10 Introduce regulatory requirements that place responsibility on AI developers and providers

AI developers and providers should be legally required to implement measures that safeguard users’ mental health and wellbeing. This includes ensuring that mental health-related outputs are evidence-informed and do not cause harm. The Australian eSafety Commission’s *Safety by Design* framework, emphasising service provider responsibility, user empowerment, and transparency, provides a useful foundation (eSafety Commission, 2026). The responsibility for safety and harm mitigation should rest with developers and providers, including obligations to support independent evaluation and research access where appropriate.

## Conclusion

AI has the potential to revolutionise mental health care. However, there are significant knowledge, policy and regulatory gaps that must be addressed before AI can be integrated safely within the Australian mental health system. Ethical oversight and peer review are fundamental to high-quality academic research. However, AI technology is advancing at a far faster pace than research and we must consider new ways to support research and respond to rapidly emerging innovations.

## Figures and Tables

**Table 1:** Current and emerging opportunities for AI in mental health.

Digital technologies, driven by AI, to improve affordability, accessibility and develop better treatments for mental health conditions	Text, voice and digital data to monitor mental health symptoms and intervene early (Shin and Bae, 2024).
	Digital AI tools to improve care navigation (Dawoodbhoy et al., 2021; Rana et al., 2024), reduce cases of missed care (e.g., inadequate monitoring, delayed medication administration; Higgins and Wilson, 2025), and support stratified care approaches for depression treatment (Delgadillo et al., 2022). AI may complement existing services that provide a trusted entry point for people who need health information, advice and connection to appropriate care and services (NSW Health, 2025).
	AI “virtual front doors” may become ubiquitous in triaging to ensure that Australians get the right care in the right place at the right time (McMahon and McInerney, 2025).
	Generative AI to offer information, support and reduce inequity of access to mental health care (Habicht et al., 2024).
	Evidence based Chatbots to provide innovative, affordable and accessible therapies (Campellone et al., 2025b; Heinz et al., 2025).
	Machine learning to develop personalised treatments for mental health conditions (Koch et al., 2024).
	AI-adaptive trials to improve the efficiency of mental health clinical trials in Australia (Huckvale et al., 2023).
	AI algorithms to develop population insights to improve mental health systems modelling (Whiteford et al., 2023) and ensure that investments result in improved mental health outcomes.
AI to support rapid development of workforce capability	AI to support training of Australian clinicians e.g., suicide prevention gatekeeper training (Holmes et al., 2025).
	AI scribes, automated scoring and generation of assessments and reports, diagnostic and decision support tools, and other technologies to reduce administrative burden for Australian clinicians (Wilson et al., 2023).
	AI has the potential to boost productivity by assisting Australian clinicians with tasks such as gathering medical information by extracting and organising data from records, tests, and digital sources, providing education, engaging clients in care, and facilitating follow-up (Higgins and Wilson, 2025).
	Remote patient monitoring and delivery of timely support (Hassan et al., 2025).
